# Thematic Analysis of My “Coming Out” Experiences Through an Intersectional Lens: An Autoethnographic Study

**DOI:** 10.3389/fpsyg.2021.654946

**Published:** 2021-04-23

**Authors:** Enoch Leung

**Affiliations:** Department of Educational & Counseling Psychology, McGill University, Montreal, QC, Canada

**Keywords:** LGBTQ, autoethnography, intersectionality, developmental psychology, identity, coming out, ethnicity

## Abstract

For lesbian, gay, bisexual, transgender, and queer (LGBTQ) youth, identity development is one of the most critical developmental task. LGBTQ youth are shown to be at risk for a variety of risk factors including depression and suicidal ideation and attempts due to how their identities are appraised in heteronormative societies. However, most LGBTQ educational psychology research have highlighted protective factors that are primarily relevant to support LGBTQ white-youth. One of the major developmental theories, Erikson’s stages of psychosocial development, has identified adolescence as the period where identity development occurs. However, through an intersectional lens, identity development appears to encompass more than adolescence but also emerging adulthood, a developmental stage not accounted for by Erikson’s stages of psychosocial development. The primary goal of this study is to seek to understand and question Erikson’s stages of psychosocial development through an intersectional lens of an autoethnography of my LGBTQ experiences. An autoethnographic approach [diary entries (*N* = 9), conversations (*N* = 12), interview (*N* = 1), social media websites and blogs (*N* = 2), and drawing (*N* = 1)] is used to understand my LGBTQ-person of color (POC) experiences of “coming out” or self-disclosure during my adolescence through emerging adulthood. Data was collected on April 2020 and spanned from 2006 through 2020 to account for the developmental period of adolescence and emerging adulthood (ages 13 through 27). Thematic analysis revealed four themes across the two developmental periods: (1) confusion and conflict between my gay and ethnic identity as a closeted adolescent, (2) my first “coming out” as a gay adolescent and “it got better,” (3) frustration arising from the internal conflict between my gay and POC identity as an emerging adult, and (4) frustration arising from external experiences with the flaws of LGBTQ community inclusivity. Results reflected a continuous theme of identity exploration and struggle through both adolescence and emerging adulthood, highlighting the need for future research to replicate similar experiences from other intersectional individuals during emerging adulthood stage, a developmental stage that is considered in between Erikson’s adolescent and young adulthood developmental stage.

## Introduction

Though LGBTQ communities have achieved recognition as a protected minority group, LGBTQ individuals, and particularly LGBTQ students (Ream, 2018), remain vulnerable to a variety of risk factors. For example, LGBTQ students face three primary categories of risk: (1) academic risks (e.g., increased absenteeism, lower academic achievement, fewer plans for post-secondary education; Kosciw et al., 2018), (2) social and emotional risks (e.g., impaired psychological well-being, higher levels of depression, lower self-esteem; Kosciw et al., 2018), and (3) behavioral risks (e.g., substance abuse and suicidal attempts; Almeida et al., 2009; Kosciw et al., 2018). In short, LGBTQ students are at much higher risk compared to their heterosexual peers. However, evidence has demonstrated that, with the support of teachers, families, inclusive school policies, and peers (Leung et al., Manuscript in preparation), LGBTQ students can thrive alongside their heterosexual peers.

The results from the current body of LGBTQ educational psychology research have inspired my own research interests within the realms of developmental psychology, education, and LGBTQ studies. The majority of research findings on LGBTQ educational psychology have framed LGBTQ students as “at-risk” population and centered on understanding LGBTQ students and their negative outcomes (e.g., depression, suicidal attempts, truancy, and low self-esteem; Russell et al., 2010; Robinson and Espelage, 2011; Kolbert et al., 2015; Kosciw et al., 2015; Earnshaw et al., 2020). As I have delved deeper into my research, several consistent research findings have struck a chord with my own identity, not as a researcher, but as an individual who has lived through the experience of being a member of an “at-risk” population, who was only deemed worthy of protection in 1996 (Ontario Human Rights Commission, 2009). This process has helped me to understand my past trauma and risks, and I have witnessed how current research reflects my past and current self as I navigate my environment as a gay, Chinese–Canadian man living in Western society. I have begun a journey of understanding and exploring the subtleties of my own identity as both an LGBTQ minority and an ethnic minority man within the context of my research. Therefore, the primary goal of this study is to seek to understand and question Erikson’s stages of psychosocial development through an intersectional lens of an autoethnography of my LGBTQ experiences. Do note a disclaimer on the use of LGBTQ as an acronym to encompass the LGBTQ community. As LGBTQ is one of the most common acronyms used to encompass the (LGBTQ) community, LGBTQ is used to denote my experiences with the community. However, although the acronym LGBTQ is used to understand the discourse of my experiences with the (LGBTQ) community, my LGBTQ identity experiences and the LGBTQ community is not a homogenous experience across the different subgroups of lesbian, gay, bisexual, transgender, and queer individuals. This is often not the case as transgender and gender non-conforming individuals experience specific paths of identity development and unique forms of stigma different from those who are lesbian, gay or bisexual (Babine et al., 2019). Whenever possible, the acronym to label a specific subgroup of LGBTQ community will be used when discussing LGBTQ experiences from different studies.

### Erik Erikson’s Stages of Psychosocial Development

During the adolescent psychosocial stage, also known as identity versus confusion stage (Erikson, 1968; Dunkel and Harbke, 2017), one of the key developmental tasks critical to LGBTQ adolescents’ identity development is to “come out” or self-disclose their LGBTQ identity to themselves and others (Bates et al., 2020). As evidenced by Dunlap’s (2016) finding, the average age at which LGB individuals “came out” or disclosed their LGB identity tended to decrease when compared across cohorts of individuals (ranging from cohorts born before 1951 to cohorts born after 1988). This may be attributable to cultural shifts within the United States as the majority of the individuals lived within the United States. The age at which individuals come out corresponds to the adolescent stage of development (Dunlap, 2016), termed as Erikson’s identity versus confusion stage. During this stage, adolescents focus on identity development, divided into identity integration and confusion, which is important for psychological wellbeing (Erikson, 1980; Rosario et al., 2011; Bates et al., 2020). Identity integration is defined as one’s acceptance of their ever-evolving identity and a desire to let others know their identities across situations (Erikson, 1980; Rosario et al., 2011). For example, LGB individuals experience the highest level of life satisfaction when they were able to be open about their LGB identity in both personal and professional environments (Lindsey et al., 2020). On the other hand, identity confusion refers to a sense of self that is inauthentic due to the continued search for a meaningful identity (Erikson, 1980; Rosario et al., 2011). LGBTQ individuals who are confused about their LGBTQ identity experience low levels of life satisfaction and struggle with low self-esteem, guilt, shame, depression, anxiety, and suicidal ideation (Beagan and Hattie, 2015).

However, though Erikson categorizes the adolescent stage as a stage for identity development, one considers whether the narrative development of LGBTQ identity spans beyond the adolescent stage of psychosocial development. Parmenter et al. (2020b) sought to understand identity development amongst young adults and found that, though participants were 20–25 years old therefore categorized as young adults in Erikson’s intimacy versus isolation stage of development (Erikson, 1982), findings highlighted LGBTQ young adults’ experiences of identity conflict and exploration. LGBTQ young adults expressed identity consolidation and conflicts in integrating their LGBTQ identity across their lives (Wagaman et al., 2016; Parmenter et al., 2020b). Particularly for LGBTQ young adults with intersecting ethnic or religious identities, their young adult experiences of identity integration were highly distressing and resulted in identity confusion (i.e., identity suppression, compartmentalization, and separation) and decreased levels of life satisfaction (Wagaman et al., 2016; Parmenter et al., 2020b).

As Erikson’s stages of psychosocial development has been understood as one of the classic stage theories of development, many researchers turn to Erikson’s stages of psychosocial development, specifically Erikson’s adolescent identity achievement versus conflict stage, to understand identity development in adolescents (Rageliene, 2016). According to classic developmental theories (e.g., Erikson’s, Piaget’s, and Kohlberg’s), they are universal and assume all individuals go through each stage. However, Erikson’s theory is considered to be increasingly irrelevant due to the changing social conditions. Though Erikson attempted to create a universalistic theory to outline the developmental trajectory across the lifespan, postmodern and critical psychologists have rejected a universal trajectory of development. Rather, they speak to the possibility for classic developmental theories such as adolescent identity versus conflict stage, a component of Erikson’s stages of psychosocial development, to have more than a singular path to achieve identity depending on the different identities and cultural context of development (Schachter, 2005). Therefore, although LGBTQ identity integration is a stage that corresponds to Erikson’s adolescent stage of development, evidence of LGBTQ identity integration suggests the development of an integrated LGBTQ identity span across multiple stages of Erikson’s psychosocial development. To further understand and question the developmental trajectory of LGBTQ identity integration and Erikson’s psychosocial development stages, an autoethnographic analysis of my LGBTQ identity development will be conducted.

### Intersectionality

Intersectionality is defined as a concept to describe how different identities such as ethnicity, sex, class, and other individual differences intersect with each other to explain differences (Crenshaw, 1991; Crenshaw et al., 1995; Cho et al., 2013). An intersectional approach highlights an individual’s need to be viewed as a whole person as opposed to a group of separate identities (Crenshaw, 1991). Moreover, intersectionality promotes an understanding of individuals as shaped by the interactions of different social identities, systems, and structures of power. It is through the understanding of the interactions of differences (e.g., race/ethnicity, gender, class, sexuality, dis/ability, and religion) that give rise to inequities and experiences of oppression, stigma, and discrimination (Crenshaw, 1991; Hankivsky, 2014). Given the context of identity development, intersectionality suggests that LGBTQ White adolescents and LGBTQ ethnic minority adolescents (or youth of color; YOC) experience their identity development differently (Hulko and Hovanes, 2018; Miller, 2018). While existing research in the field of development, such as Erikson’s adolescent psychosocial stage of development, highlight adolescence as the period for identity development, the literature surrounding intersectionality provides insight to major missing points and can be an avenue to question Erikson’s adolescent stage of development.

Intersectionality can become increasingly complex as sexual and religious identity intersect with age such that the experiences of coming out depends on contextual factors and how experiences of disclosure of LGBTQ identity changes across generations taking into account individuals’ religious and sexual identities. Rosati et al. (2020) found in their study that older adults emerged as the least disclosed group and most rejected group compared to their younger counterparts as a consequence of the stigmatized historical period in which they grew up where non-heterosexual behavior was considered immoral and condemned by all institutions. Other examples of how intersection of multiple identities and discriminations work in practice can be seen in Calasanti’s (2009) work. They found old, lesbian women to have experienced invisibility within lesbian and queer communities due to their old age. In Gibbons (2016) work, they found LGBT individuals with physical disabilities lack the accessibility to experience LGBT spaces and events. Other examples intersecting ethnicity and sexuality can be seen in other research. For example, 77% of White men disclosed their sexuality to their family compared to 51% of Asian and Pacific Islander men (Grov et al., 2006). LGB Asian individuals perceived the United States environment to be more open and supportive of their sexuality compared to their experiences in Asia (Kimmel and Yi, 2004). Therefore, an intersectional lens to understand psychological processes, such as the identity integration process (e.g., coming out), is key as Erikson’s processes can vary greatly depending on contextual variables after consideration of intersectionality (e.g., age, religion, dis/ability, ethnicity, and LGBTQ).

Interestingly, a critical review revealed scant literature on identity development for LGB-YOCs, with only one study that examined ethnic and sexual identities and how both identities developed concurrently (Toomey et al., 2017). The review revealed majority of the intersectional studies on identity development were comparative studies, with LGB White youths more likely to have accepted and integrated their identity compared to LGB-YOCs (Hunter, 1996; Toomey et al., 2017). Additionally, cultural constructs such as machismo (i.e., strong sense of masculinity) and familism (i.e., focus on family over individual needs) were prevalent in Latinx communities and found to influence their LGB identity development (Yon-Leau and Munoz-Laboy, 2010; Toomey et al., 2017). Given that most of the intersectional studies were with Black and Latinx LGB youth (Toomey et al., 2017), the autoethnography of my LGBTQ-Chinese experiences serves to provide insight into the lack of research surrounding the identity development of a LGBTQ-YOC through young adulthood.

## Methodology

Given the purpose of the study, the use of autoethnography as a tool to conduct narrative research aligns with anthropologists’ notions of the significance of the emic perspective and the importance of narrative research to understand culture (Miller, 2008). The study of identity is grounded in my constructed experiences and reflects my emergent understandings that have resulted from analyzing these experiences through the autoethnographic process. Particularly, autoethnography allows for rich and detailed data and interpretation, which has the potential to provide a first person view of their world. Given the intent of this study is to not reach generalizable conclusions, my decision to engage in this form of research was inspired by previous autoethnographic work (e.g., Miller, 2008; Rosenberg, 2017) to represent my own constructed perspectives of my experience as a gay Chinese–Canadian male to shed light on the nuanced complexities of how intersectionality questions Erikson’s psychosocial stages of development during identity versus confusion. Therefore, autoethnography is an ideal methodology positioned to question Erikson’s adolescent psychosocial stage of development (identity versus confusion) through a reflexive analysis of my intersectional LGBTQ-POC experiences of “coming out.”

### Autoethnography

*Autoethnography* is a methodology that is defined as a bridge between the observer and the observed, a “way of knowing” that acknowledges researchers’ feelings about their own experiences (auto) and the associated connection with the larger cultural (ethno) throughout the analysis (graphy; Ellis et al., 2011; O’Hara, 2018; Rambo and Ellis, 2020). Autoethnography is both a research process and a product of the approach to analyze the self and personal experiences in the context of their environment that shape one’s self, provide insight into possible alternative perspectives or theoretical lens, and connect to the wider society and further sociological understanding (Rambo and Ellis, 2020). Using autoethnography, the researcher is both the observer and the participant, both the researcher and the subject (Ellis et al., 2011). Given the intent of this study, autoethnography is one of the approaches that acknowledges and appreciates subjectivity and the researcher’s influence on research. As such, my intersectional experiences as a cisgender LGBTQ Chinese–Canadian male acknowledges the subjectivity and researcher’s influence on the research in question.

### Procedure

This paper followed specific procedures based on the methodological paper by Susan O’Hara (2018). According to her work, data for use in an autoethnography can be in the form of diaries, blogs, podcasts, or drawings. The analytical process for autoethnography comprises following qualitative guidelines for assuring that the research findings are credible, transferable, dependable, and confirmable. For the purpose of this paper, the data are primarily taken from my diary entries (*N* = 9), conversations (*N* = 12), interview (*N* = 1), interactions on social media websites and blogs (*N* = 2), and drawing (*N* = 1). Data was collected on April 2020 and data spanned from 2006 through 2020 to account for the developmental period of adolescence and emerging adulthood (ages 13 through 27).

### Data Analysis

Analysis of data will follow thematic analysis, a systematic method to identify and organize insights into meaningful themes across the data (Braun and Clarke, 2012). Following thematic analysis procedures, the data was familiarized with through the month of April 2020 and preliminary codes (e.g., internal conflict between gay and Chinese identity, negative LGBTQ experience during adolescence period) were assigned to the data to describe the content. From the preliminary codes, themes were organized across the data. The themes were then reviewed, defined, and named (Braun and Clarke, 2012). Of importance, the themes were organized with intersectionality in mind. Specifically, the preliminary codes across the various data (i.e., diary entries, conversations, interview, interactions, and drawing) were first organized according to the developmental period from Erikson’s stages of psychosocial development. Then, the preliminary codes were placed within each developmental period and analyzed through an intersectional lens (i.e., how the different data interacts between my identities and my internal and external environment). Thematic analysis aligns with autoethnography as an analytical method to allow the researcher to organize and make sense of their personal experiences with the guiding theoretical framework (Braun and Clarke, 2012). Specifically, thematic analysis will allow thematic organization of my LGBTQ-POC experiences of “coming out.” Following the methodology proposed by Liggins et al. (2013), the conversations and reflective content throughout the themes appear in italicized font and punctuation marks. Furthermore, the study was excluded from institutional review as the data represents my own narratives and no identifiable information was collected to protect confidentiality and anonymity.

### Objective

This study will apply my lived experiences as a former LGBTQ-POC adolescent and current young adult to map onto Erikson’s identity versus role confusion stage. Through autoethnography, I hope to analyze my “self” identity and journey in the context of identity development, acknowledging the intersection of my two primary minority identities that fundamentally shape who I am, to provide insights into alternative perspectives and possible gaps in Erikson’s identity versus role confusion stage.

## Experiences and Reflections

Following thematic analysis of data spanning from 2006 through 2020, findings were organized under four themes that underlined the developmental trajectory of Erikson’s identity versus role confusion stage of psychosocial development. The first theme underlies the beginning of identity development during adolescence and the conflict and confusion with both my LGBTQ and ethnic minority identity. The second theme conciliates Erikson’s identity acceptance through the disclosure of my LGBTQ identity to myself and others and subsequent support and conflict as a result of coming out. The third theme explores an intersectional conflict between the acceptance of my LGBTQ identity and my ethnic minority identity. The fourth theme describes the eventual internal consolidation of both LGBTQ and ethnic minority identity but redirecting my identity acceptance toward a stage of identity activism due to the external frustration toward inclusion of LGBTQ ethnic minority individuals in the LGBTQ community.

### Theme 1: Conflict and Confusion in the Initial Stages of Erikson’s Adolescent Identity Development

#### Subtheme 1.1: Stigma and Discrimination at School

I knew I was “*different*” since elementary school. During my adolescence from 2006 through 2010, I realized through constant knowledge-gathering in Hong Kong and other Eastern countries that my “*kind*” was not accepted and was looked down upon. When I asked prompting questions to my family and peers, such as “*What are your thoughts with this Hong Kong singer coming out as gay or feminine*” (Leung, conversation, August 28, 2009) or accidentally revealed my computer background of a male, Caucasian celebrity, I gathered information that made it clear that being gay was a negative thing. My peers and family responded, “*don’t talk about him, no one talks about him anymore*” (Chinese, 16–17 years old, conversation, August 28, 2009) and “*why do you have a white guy as your computer background?*” (Chinese, early 40s, conversation, July 13, 2010). As I navigated through the hallways of my school in Hong Kong, I was similarly harassed for being “*different*” compared to my Chinese peers. Peers mentioned, “*Why do you look like that*…*so fat and round?*” (Asian, 12–13 years old, conversation, September 03, 2006), “*You always play videogames and card games, what a nerd, and a stupid one too*” (Asian, 13–14 years old, conversation, October 07, 2007). As schools in Hong Kong had classes differentiated by ability, I did not fit in with my peers due to my grade and class I was placed in: English Set 3 of 3; Math Set 4 of 6; Chinese Set 2 of 2; French Set 2 of 2. Being outcasted for my academics, appearance, and hobbies, I kept to myself to not be further outcasted for my attraction to men.

As I experienced high school abroad in a rural Canadian city in Ontario, the negative climate persisted where LGBTQ individuals were treated as pariahs. While my school friends perceived their classroom as safe and engaging, I perceived it as distant and hostile due to teachers putting down the LGBTQ community, either due to a lack of knowledge or internal beliefs about the LGBTQ community: My health/science high-school teacher said, “*If you’re gay, you’re going to get HIV/AIDs*” (Caucasian, early 30s, conversation, March 27, 2009), and my peers agreed. I felt awful, even just reliving the memories. Walking along the hallways of high school, I would hear phrases from peers “*what are you looking at, you queer faggot?*” (Caucasian, 15–16 years old, conversation, February 21, 2009) when I was looking at my male peers in class and “*that’s so gay*” (Caucasian, 15–16 years old, conversation, September 17, 2009) as a derogatory response to the English movie *She’s the Man* for an English class. As a closeted adolescent attempting to make sense of my own identity as aligned with Erikson’s adolescent psychosocial stage of development, I felt many negative emotions for having homosexual thoughts: confusion, self-loathing, guilt, shame. From my high school experiences in rural Canada, I understood that the LGBTQ community was outcasted. From my cultural experiences in Hong Kong, I understood that Chinese culture also outcasted LGBTQ individuals as unnatural.

#### Subtheme 1.2: Emerging of an Intersectional Awareness

Though I was a Chinese–Canadian gay adolescent, I was confused where I stood in my school and community, where LGBTQ identities were not accepted.

According to Gato et al. (2020),

During recess and if I was alone it was common to be called gay (Male, 19, gay, cisgender)…there was a lot of prejudice, I lost all my “friends,” my class put me aside completely (Female, 19, lesbian, cisgender)…my colleagues used to strangle my neck in 7th grade because I was gay (Male, 16, gay, cisgender)…. A school employee talked about our relationship to my girlfriends’ family and ruined our relationship (Female, 16, lesbian, cisgender)…. In my school, with my colleagues, it’s the only place where I don’t feel comfortable revealing my sexuality (Female, 15, pansexual, cisgender).

In the identity versus role confusion stage, adolescents examine their identity and understand who they are and want to be as they situate themselves within society (Erikson, 1968). If adolescents are unsupported or left confused and unable to establish a sense of identity within society, it can lead to role confusion, leaving individuals unsure about themselves and their place in society. Additionally, adolescents who perceive a lack of LGBT role models and cultural reference points are additional factors that compromise the identity integration process (Institute of Medicine, 2011). For example, my high school experiences led me to internalize the belief that being gay is a sin. Though I was not religious, positioning myself in a religious high school in rural Canada did not positively influence my identity consolidation. Classroom material taught me that LGBTQ individuals are at risk for HIV/AIDS. High school peers were using LGBTQ terms in a derogatory form. My high school experiences echo Snapp et al.’s (2015) student perspectives I perceived in school spaces where I was unable to excel in my high school due to the lack of safety and comfort. Though I was not openly gay during high school, my feminine characteristics led peers to believe that I was a *queer*, a *faggot*. Walking through the school environment, I was afraid of being harassed for my physical characteristics and mannerisms. This did not create a space comfortable to learn and be authentic. My solution, however, led me to be in a position of power where such verbal harassment is avoided, believing that being in a position of power can minimize verbal harassment.

It is clear how my negative mental health outcomes were in part due to the lack of integration of my sense of self. The disgust and internalized homonegativity that I harnessed toward my feelings for and attraction to men contradicted my personal beliefs and the values that I was initially raised to hold. My personal beliefs coming from an ethnic Chinese identity brings about the concept of filial piety and collectivism (Lieber et al., 2004) which are systems of beliefs that are exposed to individuals from collectivistic cultures like Hong Kong or Japan (Yeh and Bedford, 2003). Filial piety is a moral-based system where individuals have learned to respect and obey the wishes and expectations of their parents and to defer or ignore their own personal needs. As such, being gay or otherwise “different” was not acceptable in the Chinese community due to the fact that LGBTQ identities are perceived as shameful and atypical, bringing shame and disrespect to the family (Quach et al., 2013). On the other hand, being gay or “different” in a Western environment, such as my high school in rural Canada, was less about shame and disrespect to my family but more targeted to my lack of individual ability to be authentic in my personal identity that is otherwise being harassed and labeled as weird, feminine, and not a ‘real’ guy (Jewell and Morrison, 2010).

However, my ethnicity was still part of my identity. The push and pull between my Eastern values and beliefs from back home in Hong Kong and my understanding of Western values in Canada about being true to one’s self complicated this developmental task. As a result, I felt compelled to “throw” or dissociate one part of my identity. Throughout my adolescent years, my anxiety, depression, and suicidal attempts was the result of struggling to understand how the two identities were perceived in our societies. I came to see that my initial feelings of internalized homophobia and xenophobia, self-disgust, and guilt are aligned with Erikson’s identity versus role confusion stage, as my two identities were in conflict. Erikson’s adolescent stage of development posits that identity synthesis (making sense of self over time across situations) and consolidation (dynamic interplay between identity synthesis and confusion resulting in healthy development) are associated with low levels of internalizing (e.g., depression, anxiety, and suicidal ideation and attempts) and externalizing symptoms (e.g., peer conflicts and delinquent behaviors; Hatano et al., 2018) whereas identity confusion is associated with high levels of both. My decision to dissociate and forego understanding my identities lend accurately to the identity confusion aspect of Erikson’s identity development. However, Erikson’s identity development stage becomes less clear when taken into account two identities.

### Theme 2: Disclosure of LGBTQ Identity – Consolidating Erikson’s Identity Development

#### Subtheme 2.1: Implications of Stigma on Mental Health

As I arrived in Montreal during 2010 to begin my undergraduate studies, my identities continued to be in conflict. I engaged in non-suicidal self-injurious behaviors (i.e., cutting) and partially withdrew from my classes due to how LGBTQ identities were appraised in societies and the difficulty to assess my identities against my cross-cultural experiences.

“*I can’t take this anymore. I don’t want to die but this is insufferable. Although I love my family and want to take care of them, I think they have to accept me for who I am. Either I lose my family or I die.*” (Leung, diary entry, October 12, 2010).

#### Subtheme 2.2: The Crucial Role of a Supportive Context

I then made the decision to “come out” after realizing that Montreal, Quebec was more accepting and inclusive than my religious high school in rural Canada and my family and home culture in Hong Kong. I felt I had to self-disclose my sexuality to feel comfortable in my own skin. The doubt, guilt, and self-loathing feelings I garnered within myself was too much and those feelings felt overwhelming. Compared to rural Canada or Hong Kong, where both locations lacked LGBTQ presence and visibility in the community, the streets of downtown Montreal and the LGBTQ-dedicated *Village* in Montreal showed positive representation and openness to LGBTQ individuals. I was able to observe openly LGBTQ individuals in Montreal and saw visible symbols and signs of LGBTQ inclusivity, such as a rainbow triangle labeled as “Safer Spaces” or rainbow flag outside different business stores. The visual evidence that surrounded Montreal aligned with previous research that highlighted adolescents’ perceived importance of LGBTQ visual cues (rainbow flag and pink triangle) as indicative of possible safe and inclusive spaces (Porta et al., 2017a). Immersed in the Montreal community and spaces, I felt safe knowing I had supportive friends and local community spaces that were accepting of the LGBTQ community. I finally felt safe being able to identify who I truly was.

#### Subtheme 2.3: Reactions to Coming Out

However, self-disclose to my family was met with a response commonly found in previous literature surrounding LGBTQ adolescents’ coming out experiences. Roe (2016) found that LGBTQ students reported unsupportive reactions from their parents, ranging from refusing to acknowledge their child’s disclosure to telling their child they will go to hell. I called my mother, “*Mom, I’m gay* [*long silence and then phone hangs up*]” (Leung, conversation, November 02, 2010). Over the holiday break, my mom came back to the coming out conversation, “*Can you turn back straight for me?*”, and my dad attempted to reason with me, “*Can you turn back to being straight? Mom is feeling very suicidal and is blaming herself for you being gay*” (Leung, conversation, December 21, 2010). My parents’ reaction to my self-disclosure aligned with previous research on family support during self-disclosure. Though I self-disclosed to my family and friends, my level of comfort in disclosing that I was gay as a Chinese–Canadian adolescent was low. I promised my parents that I would go back to “*being straight*” to ease their concerns as I did not want to let my parents down, attributable to Chinese cultural influences and its emphasis on familism (Chan et al., 1997; Campos et al., 2014; Lu, 2020). Familism is an ideology that puts the priority to family and avoid bringing shame and disrespect to the family (Bedford and Yeh, 2019). The collectivistic culture that is well documented in literature surrounding Chinese traditional culture emphasizes the principle of respecting seniors and prioritizing interpersonal obligations over individual needs (Hu et al., 2018). Knowing my Chinese family was still uncomfortable with the idea of me coming out as gay, I was caught in between identifying as a closeted Chinese and an openly gay Canadian.

Following Erikson’s identity versus role confusion stage, by coming to terms with my LGBTQ identity and disclosing my gay identity to others, this can be understood as Erikson’s identity consolidation where I would experience low levels of internalizing (e.g., anxiety, depression, and suicidal attempts) and externalizing (e.g., truancy) symptoms (Hatano et al., 2018). This was true. By coming to terms with my LGBTQ identity, the LGBTQ education research (e.g., Craig and McInroy, 2014; Kosciw et al., 2015; Russell and Fish, 2016; Human Rights Campaign Foundation, 2018), suggests that I should experience a higher level of psychological wellbeing and many other positive factors, such as academic success and improved peer relations. As I perceived my environment to be safe to disclose, I publicly disclosed my gay identity as I was living independently from my family and had a higher level of confidence. I felt more comfortable in my skin because I had several close friends who supported and accepted me. However, when understanding my self-disclosure through an intersectional lens, questions arise whether I truly consolidated my ethnic and LGBTQ identity with my overall sense of self or whether I chose to ignore my ethnic identity and importance of familism in my culture. Through an intersectional lens, I came to accept myself as a gay man and reject my ethnic identity as I understood my family and the Chinese community to be unaccepting of LGBTQ individuals.

The experience aligns with the literature on conflicting identities. Specifically, Koc and Vignoles (2016) mentioned the concept of bicultural identity integration which consists of two bipolar components of identity integration, *blendedness versus compartmentalization* and *harmony versus conflict.* The first component, *blendedness versus compartmentalization*, represents the degree of differences or similarities perceived between the two identities (Koc and Vignoles, 2016). The second component, *harmony versus conflict*, represents the level of tension or stress on the compatibility between the two identities (Koc and Vignoles, 2016). According to the concept of bicultural identity integration, my identity development experiences with both my LGBTQ and ethnic minority identity is then more compartmentalized and in conflict with each other due to the differences and tension between the two identities. Therefore, with Erikson’s identity versus role confusion stage, my identity development had finished after I consolidated my LGBTQ identity. Logically, I would move toward Erikson’s young adulthood psychosocial stage of development, intimacy versus isolation, defined as moving toward achieving healthy, fulfilling relationships (Erikson, 1980, 1982; Kacerguis and Adams, 1980; Gold and Rogers, 1995; Starks et al., 2017). On the contrary, when identity development is understood through an intersectional perspective, the lack of ethnic identity consolidation with my LGBTQ identity is indicative that my identity development is incomplete.

### Theme 3: Understanding Internal Frustrations of Identity Through an Intersectional Lens

#### Subtheme 3.1: Changing Perspectives on LGBTQ Issues

“*I’m confused. I’m supposed to be happier since I’m openly gay. Why do I feel like I still don’t belong? Why do I feel so isolated? Should there not be positive experiences within the LGBTQ community being welcoming and a safe space?*” (Leung, diary entry, September 21, 2015)

Due to my negative experiences as a LGBTQ adolescent in a negative school climate and unsupportive family, I pursued graduate studies to understand how to support LGBTQ adolescents and minimize their risks. However, I was overwhelmed with the number of articles focusing on LGBTQ adolescent and young adults’ struggles (e.g., depression, substance abuse, suicidal ideation and attempts, isolation, stigmatization, and lower educational aspirations; Russell et al., 2010; Robinson and Espelage, 2011; Poteat et al., 2013). My goal was to flip the narrative from the negatives to the positives of being a LGBTQ young adult.

“*I am sick and tired of hearing all the negatives about myself; there must be something good out there about being a LGBTQ individual. Shouldn’t coming out make it all better like what Dan Savage’s book said? That it will get better? What exactly gets better? For who? And for what?*” (Leung, diary entry, August 10, 2016).

“*Identifying as LGBTQ must be difficult; they’re all such a mess and have a rough life*” (Caucasian, early 20s, conversation, April 15, 2016). While I was writing my comprehensive exam paper, I was fed up with research that continuously mentioned the vulnerability of LGBTQ youth and the LGBTQ identities due to their appraisal in a heteronormative society. “*I’m worthless*, *I’m just another statistic*” (Leung, diary entry, December 02, 2017). I did not really understand where this feeling was coming from. “*They don’t really get me. I’m more than this. Were the findings through other published articles hitting a nerve close to home?*” (Leung, diary entry, December 02, 2017). As I relived my past thoughts of suicidal attempts while writing my comprehensive exam, I wondered whether “*it gets better*.”

#### Subtheme 3.2: Adopting an Intersectional Lens

From 2018 onwards, during one of the education classes I taught as a course instructor, several students raised an interesting point that I did not previously take into account in my research: A student asked, “*How would adolescent development change if culture was taken into account?”* (Asian, undergraduate student, conversation, February 06, 2019). A second student followed up with another question: “*I agree. As a Chinese–Canadian student, I don’t fully understand the importance of students to feel comfortable and safe in their classroom to learn*” (Chinese–Canadian, undergraduate student, conversation, February 06, 2019). Taking a Chinese cultural perspective, the comment may stem from the Confucian principles that place a high value on education and learning, with the belief that education is for everyone and hard work can compensate for a lack of ability. Most importantly, for Chinese individuals who are influenced by Confucianism, an ancient Chinese belief system, they believe learning is a moral duty and studying hard is a moral responsibility to the family, irrespective of feeling safe and comfortable in a classroom (Wang, 2006). On the other hand, there are considerable literature that emphasizes the importance of safe spaces for students to engage in classrooms based on samples from Western societies (Theobald et al., 2017). This interaction revealed a compelling intersection that I originally did not account for in my research.

Through my teaching and research, I reflected on my understanding of my own identity and began to wonder whether my vulnerability and frustration toward LGBTQ research in educational psychology was a result of the lack of intersectionality. As mentioned in previous themes, my ethnicity and LGBTQ identity were not consolidated. Positively appraising my LGBTQ identity meant that I was foregoing my ethnic minority identity, and vice versa. The negative experiences of struggling to find a balance between my two minority identities with a lack of support and positive experiences to support my identity exploration contributed to my negative mental health outcomes (i.e., suicidal ideation and attempts). My rejection to my Chinese identity mentioned in Theme 2 contributed to my lack of acknowledgment of my POC identity. Being immersed in Chinese culture while I was in Hong Kong, I experienced negative, homophobic experiences that led me to detach myself from my Chinese identity. Aligned with McCormick and Barthelemy’s (2021) research on acknowledgment and acceptance of LGBTQ youths’ identities through observing LGBTQ representation in their community, though I did not feel safer while I was in a religious high school in rural Canada due to the lack of LGBTQ representation and conflict often observed between religion and LGBTQ (Dahl and Galliher, 2012; Beagan and Hattie, 2015; Gibbs, 2015), diverse LGBTQ representations in Montreal led to a reflection on my own intersectional identities.

#### Subtheme 3.3: Revisiting Erikson’s Theory

As a 26-year-old gay male, I would correspond to Erikson’s young adulthood stage of psychosocial development, intimacy versus isolation stage (Erikson, 1982). However, analyzing the identity conflict through an intersectional lens (the dissociation of my ethnic minority identity with my LGBTQ identity due to a negative cultural environment), my identity development appears to have been incomplete and is now coming into question. However, identity conflicts at the age of 26 does not accurately map onto Erikson’s young adulthood stage (ages 18 through 40). This ties to evidence supporting modern developmental stage theorists that incorporate emerging adulthood in between adolescent and young adulthood stages (Arnett, 2000, 2004).

Though not in Erikson’s stages of psychosocial development, emerging adulthood is a novel stage proposed by Arnett (2000, 2004). Emerging adulthood consists of five overarching developmental milestones: (1) further *identity exploration*, (2) *instability* (e.g., jobs, relationships, and residence), (3) optimism toward *possibilities*, (4) focusing on *self*, and (5) acknowledgment of *feeling-in-between* where individuals in this stage do not feel like adolescents but not ready to be a young adult. Though my identity development persisted from adolescence, at the age of 13, identity development was not completed prior to entering young adulthood (age 18). Without taking into account my ethnic identity, my initial self-disclosure and acceptance of my LGBTQ identity could be understood as a form of premature identity formation (Erikson, 1980). I did not fully understand my identity due to the intersectionality of my LGBTQ and POC identities. Therefore, at the age of 26 where I continued to consolidate my intersectional identities, my psychosocial development aligns with Arnett’s *emerging adulthood* stage.

“*Am I currently only exploring my identity and understanding how I fit in not only as a gay man, but a gay, Chinese–Canadian man? Isn’t identity exploration in the adolescent stage of development? I thought it was as simple as coming out, accepting who I am as a gay individual and moving on from the identity piece?*” (Leung, diary entry, March 09, 2019).

#### Subtheme 3.4: Coming to Terms With the Intersection of Identities

Prior to my intersectional understanding, my past adolescent self and current young adult self faced stressors from two communities: (1) my Chinese–Canadian identity and (2) my LGBTQ identity. Based on an intersectional understanding of my identity development, my negative experiences and mental health outcomes (e.g., internalized homophobia, depression, and suicidal ideation and attempts) made more sense. My ethnic-specific stressors (e.g., focus on familism, family wellbeing, and familial piety) were directly related to my LGBTQ specific stressors (e.g., being authentic to myself, coming out as gay). I realized that I had to conceal the LGBTQ part of my identity to appease my ethnic-specific stressors. Meanwhile, my LGBTQ-specific stressors urged me to explore and understand my LGBTQ identity and the experiences of being a gay youth. Appeasing one minority-specific stressor further added stress to the other minority-specific stressor.

Though I accepted myself for who I am as a gay Canadian male, I was confused whether I could accept myself as a gay Chinese–Canadian male. The ethnic-specific stressors of familism and cultural scripts led me to believe that I was not able to be a gay Chinese–Canadian male. Though I was openly gay in Canada, I still held the promise to my parents that I *would turn back to being straight.* In November 2018, my friend engaged in self-harming behaviors due to an unfortunate relationship breakup where his partner’s parents told him to turn back to being straight or be disowned from the family. Though I was there to console him, I was having difficulty regulating my emotions and wanted the support of my family. “*I can’t do this anymore. I’m happy here in Montreal, being able to be myself, but I can’t ignore my family anymore*” (Leung, diary entry, November 10, 2018).

“*Mom, about the promise I made in 2010, I can’t do it. I was never able to turn back to being straight. It’s not possible and I’m getting too tired for this. Listen, mom, I was in a dark place back in 2010 and was suicidal for awhile. I wanted to be a good son and make my family proud but I just can’t. I’m still just as conflicted and lost because I embrace myself being gay but I want my family to support me as well. I’m not only gay but a gay Chinese–Canadian and I want to acknowledge that but knowing how Chinese culture is with LGBTQ people, I just need to at least know that my family supports me and that I’m not outcasted by my Chinese background.*” My mom replied, “*Of course, I knew all along and it was just tough during those times. You know that I love you so much and support you no matter what. It’s okay now. I’m okay with it.*” (Leung, conversation, November 10, 2018).

As a LGBTQ-POC emerging adult, integrity with my family, emphasized in Chinese culture (Chan et al., 1997; Campos et al., 2014; Lu, 2020), was critical to an intersectional identity consolidation. After having the second self-disclosure conversation with my family, I was finally accepted by my family and was able to internally consolidate both my ethnic and LGBTQ identity. I experienced many positive emotions in being able to show my true self to my family. However, though I was able to internally consolidate my intersectional identity conflict, there appeared to be another layer of identity frustration, focused on the external representation of LGBTQ-POC communities.

### Theme 4: Internal Acceptance and External Frustration Toward Inclusion in the LGBTQ Community

#### Subtheme 4.1: Isolation From the LGBTQ Community

Though I have come to consolidate my internal conflict between my LGBTQ and ethnic identity (i.e., accepting myself as gay while being mindful of my family’s feelings and wellbeing [familism]), my stressors extended beyond internal identity consolidation between my LGBTQ and Chinese identity but also external community acceptance as a LGBTQ-POC emerging adult.

*“But I don’t understand. I now have supportive friends, family, peers, and colleagues. My work environment is inclusive. What was it exactly?*…*No. I’m simply inserting myself into environments that are inclusive and safe spaces, but not about my identity. I am a minority within a minority. I am part rainbow and part colored.*” (Leung, diary entry, August 16, 2019).

Intersectionality was the answer to fully unpack and understand my vulnerability and frustrations. Having my ethnicity brought to the forefront as a Chinese–Canadian made it clear that I was not fully represented in the LGBTQ youth research.

While I had self-disclosed and accepted myself for who I was as a LGBTQ-POC emerging adult, I experienced external stressors imposed upon my intersectional identity. I realized that I did not feel fully included in the LGBTQ community. My sense of belonging did not fully lie in the LGBTQ community due to my ethnicity. “*Then who am I? What am I supposed to feel?”*(Leung, diary entry, August 16, 2019). The research aligned with previous intersectional research where LGBTQ-POC individuals experienced racialized discrimination and feelings of invisibility and exclusion within the LGBTQ community (Cisneros and Bracho, 2020; Parmenter et al., 2020a).

“*What am I feeling? I thought being openly gay was all happy and positive? But like my students have mentioned, I forgot the importance of intersectionality. I am also Chinese–Canadian. How did that affect me when I was a gay youth and now a gay emerging adult?*” (Leung, diary entry, August 16, 2019).

**FIGURE 1:**
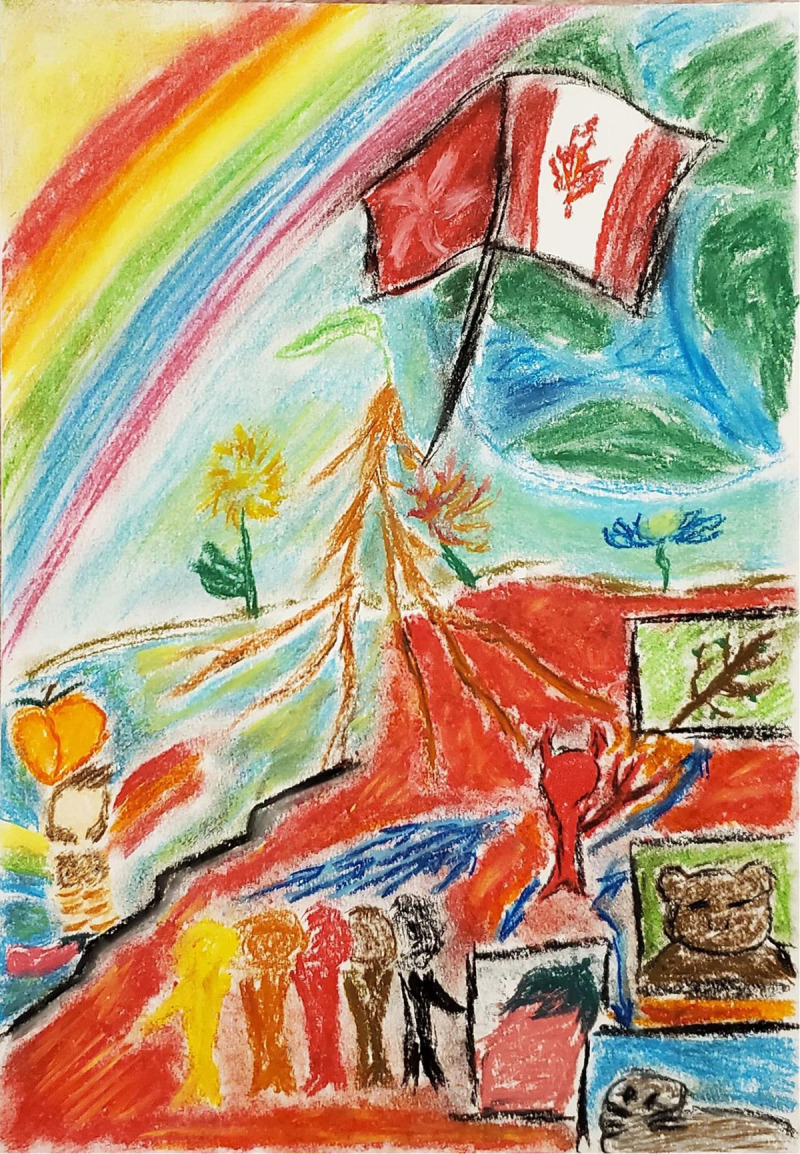
Abstract interpretation of LGBTQ+ research and intersectionality. Above the ground depicts a positive outlook to LGBTQ and ethnic minority representation. Underground depicts the division and categorization between the ethnicities.

The drawing (see Figure 1) captured how I fit in as a gay Chinese–Canadian man. It made sense. The LGBTQ education research made sense to me, and through it I understood the rainbow part of myself, that I “belong” to the “rainbow” community as a gay man. This reflection provided an intersectional lens of understanding the LGBTQ research in education and psychology, how my negative experiences and stigma arose once I engaged with the larger LGBTQ community, and how it did not “get better” (Savage and Miller, 2011).

Although I accepted myself for who I was and my school friends and coworkers accepted me, I realized I was not fully accepted by the larger LGBTQ community. The pastel drawing (Figure 1) is a creative representation that depicts my feeling that it was impossible to parse apart my sexual minority and ethnic minority identities. On the surface, evidence has demonstrated that society is becoming more inclusive and accepting of LGBT individuals (e.g., Magrath, 2020) and that Canada as a whole is increasingly inclusive of ethnic minorities (Ghosh et al., 2019). However, when I reflect on my own intersectional experiences, the narrative is more complex.

#### Subtheme 4.2: Fetishization of LGBTQ Chinese Bodies

My LGBTQ-specific stressors shifted from the developmental task of self-disclosure or “coming out” in a safe environment in school to receive general acceptance to seeking acceptance within the larger LGBTQ community by adhering to the restrictions of the stereotypes and sexual scripts of being a gay Chinese man. As I dug deeper under the surface of my drawing, I saw the negative experiences that I could not form into words, but wanted to express: how LGBTQ youth education research emphasizes the benefits of being out, but does not address how to prepare and inform LGBTQ youth about the stigma and discrimination happening within the LGBTQ community itself. I felt that I was being categorized by a higher being, that since I was Chinese, I must be in the “twink” category: a gay slang term to describe a gay young man whose physical attributes have little to no body hair, with a slim physique, common for gay southeast Asian cultures (Eguchi and Long, 2019). I had a feeling of ingroup–outgroup association, a feeling that the majority of the experiences in LGBTQ youth education research surround the experiences of LGBTQ White youth.

“*I realized that I was different even within the LGBTQ community. I had to fit into this category of being slim. But I’m not slim. No wonder I don’t fit into the LGBTQ community. At least*…*not fully, not until I fit into how LGBTQ Asians are expected to look like.*” (Leung, diary entry, April 09, 2020).

In an interview I conducted with a fellow LGBTQ scholar, he further reinforced my feelings and agreed that there was a certain issue within the LGBTQ community.

“*Yeah*…*several other participants have actually talked about body image within their interviews and talking about how like there’s this kind of – how am I trying to put it? –there’s like an association between ethnicity and body image and kind of like this fact that many LGBTQ people of color are often fetishized for body image, as well as just like their race or ethnicity’s fetishized as well*” (Caucasian, mid to late 20s, interview, 2020).

This interview touched upon exactly what my painting expresses. First, I perceived a divide between the LGBTQ Caucasian community and the LGBTQ-POC community and felt that those who were LGBTQ-POC were pre-determined by the larger LGBTQ community to have and follow social norms (attitudes, behaviors, and physical appearances) that aligned with their ethnicity. If I did not follow or fit into the social norms, I would be stigmatized, left aside or outcasted, and prevented from joining the happy, positive side of being a LGBTQ individual with stereotypical features. Previous research has recorded similar experiences among LGBTQ-POC individuals. In such studies, LGBTQ-POC young adults reported receiving comments such as “How can you be asexual? You’re Latino;” “You are supposed to be this spicy sexual person;” “You should be able to seduce a man just by staring him down” (Gonzalez, 2019). These pre-existing stereotypes and social norms within the LGBTQ community that portray LGBTQ-POC individuals as adhering to specific categories of body expectations and sexual scripts is a cause for concern regarding identifying and self-disclosing as a LGBTQ-POC emerging adult.

“*Something doesn’t feel right. We [LGBTQ-POC] come out so that we can be true to ourselves and accept our identities. Generally, in schools, then, we are accepted. This is true. In these last few years, I feel very welcome and accepted as a LGBTQ-POC young adult. I do experience a sense of belonging, acceptance from myself and others, and an overall positive psychological wellbeing. However, once this circle expands to the larger community, particularly within our own LGBTQ community, we are discriminated against because we don’t look or act a certain way? Is this the vulnerability I am feeling: that I can’t find happiness in either the Chinese–Canadian community or the LGBTQ community? Do I not have a community to belong to unless both of these identities are left behind or in communities where these identities are not highlighted? What do I have to do to feel like I am accepted for who I am, as a whole human, with a fully consolidated identity?*” (Leung, diary entry, April 20, 2020).

While I have been happier and possess a higher level of psychological wellbeing after accepting myself as a LGBTQ-POC individual, I have also suffered in my level of confidence, self-esteem, and psychological wellbeing and experienced increased feelings of anxiety, depression, body dysmorphia, and other mental-health concerns due to the confinements of being a LGBTQ-POC individual. My narrative experiences align with other LGBTQ-POC individuals on online communities. Blog articles (e.g., Kumar, 2020) and online LGBTQ forum communities (e.g., Ladblokes, 2020) similarly detail the issues surrounding racism and stereotypes within the gay and bi community that lead to anxiety and depression. Though self-disclosure of LGBTQ identities in schools can result in positive psychological wellbeing through an open and safe school space for identity exploration, LGBTQ-POC youth later grow up and realize that, in the larger society where dating and relationships occur, they are more likely to be discriminated against, fetishized, and made not to feel welcomed in the LGBTQ community unless they fit into certain social categories, placing them at a risk for anxiety and depression (Balsam et al., 2011).

### Gaps and Future Research

Throughout the self-reflection on my experiences, several gaps and questions arose that future research could explore. First, the educational psychology research concerning the translation of support for identity exploration among LGBTQ-POC youth in schools to LGBTQ-POC emerging adults facing external struggles of stereotypical expectations and sexual scripts in the larger LGBTQ is an avenue for future research. Acknowledging the developmental stages between adolescence and young adulthood reveals that supporting LGBTQ-POC youth in schools can move beyond fostering a sense of belonging and safety in school environments for identity exploration but also to learn to understand the realities of intersectional identities and how intersectional identities can affect individuals’ sense of belonging and self-acceptance within the LGBTQ community. Porta et al. (2017b) found results that LGBTQ-POC adolescents, compared to white LGBTQ adolescents, revealed a lack of engagement in LGBTQ-related social support systems. The discrepancy inquires whether the gap is due to identity struggles between one’s ethnic minority identity and LGBTQ identity, as reflected in my initial themes that mentioned the push and pull of my internal struggle with my cultural values and beliefs. The question remains whether schools can be a space to support LGBTQ-POC adolescents in consolidating their intersectional identities toward self-acceptance and prepare them to understand the external struggles in the LGBTQ community, such as the stereotypes and scripts, that can affect further identity exploration during Arnett’s (2000, 2004) emerging adulthood stage.

Second, the results reflected a continuous theme of identity exploration and struggles through both adolescence and emerging adulthood. Though the identity developmental task happens during Erikson’s adolescent developmental stage, identity is a key issue throughout Erikson’s adolescent developmental stage. However, previous research by intersectional theorists (Torres et al., 2009; Rogers, 2018; Velez and Spencer, 2018; Duran and Jones, 2020) has argued that there are discrepancies in Erikson’s stages of psychosocial development and that identity consolidation may not fully occur until individuals reach emerging adulthood stage of development when they further explore and make commitments to understand their identity (e.g., Jones et al., 2012; Jordan and Tseris, 2017). Results from my intersectional experiences merit future research to replicate similar identity exploration and struggles from other intersectional individuals during *emerging adulthood* stage (Arnett, 2000, 2004), a developmental stage that would be considered in between Erikson’s adolescent and young adulthood stage of development.

## Conclusion

As the purpose of this study is to understand and question Erikson’s psychosocial stages of development through my intersectional experiences, the experiences provide insight to better understand identity development beyond Erikson’s adolescent stage and include Arnett’s stage of *emerging adulthood*. As a LGBTQ-POC individual, my lived experiences do not fully align with current LGBTQ educational psychology research. Though identity exploration is key according to Erikson’s psychosocial stages of development, my experiences highlighted differences in the adolescent [identity versus role confusion] stage after accounting for intersectionality.

According to Erikson’s stages of psychosocial development, identity exploration should be completed during the adolescent developmental period. However, I continued to undergo intersectional identity development and self-reflection through emerging adulthood. At the surface level, I was struggling with my identity consolidation between my POC and LGBTQ identities, reflecting the adolescent stage of identity versus role confusion. However, the struggle of identity consolidation and self-reflection stemmed from external influences as opposed to internal struggles of who I considered myself to be. My experiences can be conceptualized as external identity consolidation, as establishing an intersectional identity within the LGBTQ community proved to be difficult due to the expectations and stereotypes that LGBTQ-POC emerging adults are expected to abide by.

I felt a sense of isolation and a lack of belonging to the LGBTQ community even after my struggle of self-acceptance of my LGBTQ-POC identity. Rather, it resulted in similar negative mental health outcomes as I experienced during my adolescence: anxiety, depression, hopelessness, isolation, frustration, and body dysmorphia.

As I undertook a journey of self-reflection through conversations, blog posts, drawing, and teaching, I was able to understand and untangle my intersectionality as a LGBTQ-POC individual. However, the untangling of my intersectional identities revealed that it does not simply “get better” as many LGBTQ activists and allies say. Instead, accounting for intersectionality further complicates the developmental process. Identity development, as proposed by Erikson’s adolescent stage of psychosocial development, does not only happen during the adolescent stage but also spans beyond adolescent and into emerging adulthood, a stage in between Erikson’s adolescent and young adulthood stages.

As a gay Chinese–Canadian male researcher in developmental psychology, I wanted to change the narrative that I was reading in journal articles on LGBTQ youth. I felt hopeless and powerless in the face of many articles highlighting the risks faced by LGBTQ adolescents and young adults. My original purpose was to change the narrative through leveraging my own lived experience and to expand the narrative beyond the negative statistics. As I reflected on my identity exploration and experiences, my experiences did not fully align with existing LGBTQ research in developmental and educational psychology. My journey culminated in a drawing that manifested the complex and intersectional relationships between my LGBTQ and POC identities. On the surface, coming out is often celebrated and many people (e.g., Dan Savage) maintain that it gets better. However, the divide between LGBTQ and POC identities highlights a sorting system, categorizing LGBTQ-POC individuals based on their ethnic minority identity, which assigns to them a specific set of stereotypical expectations including behaviors, body type, and sexual scripts (Darling-Hammond, 2018; Worthen, 2018; Eguchi and Long, 2019). Thus, in contradiction to LGBTQ educational research to date (Poteat et al., 2013; Craig and McInroy, 2014; Russell et al., 2014; Fish, 2020; MacDonald, 2020), self-disclosure or “coming out” may not necessarily result in improved psychological wellbeing or other positive factors.

The social and policy implications of the thematic analysis of my experiences highlight the important role of schools to support LGBTQ-POC adolescents. Though there is an increasing number of literature that understand the experiences of LGBTQ adolescents in schools, there is limited research that highlights challenges and practices to support LGBTQ-POC adolescents (Gonzalez, 2019). Across all racial-ethnic minority LGBTQ adolescents (i.e., Black, Latinx, Native and Indigenous, Asian American, and Pacific Islanders) who experienced harassment in school, the majority of them (73.9% of LGBTQ Native and Indigenous adolescents, 67.4% of LGBTQ Asian American and Pacific Islander adolescents, 63.5% of Latinx LGBTQ adolescents, 62.9% of Black LGBTQ adolescents) did not reach out to school staff members for support due to the perception that they do not think that staff would do anything about the race-based and LGBTQ-based incidents (Truong et al., 2020a, b; Zongrone et al., 2020a, b). The lack of safe adults and teachers to reach out to for support due to their intersectional identities may deter them from trusting school staff to disclose their LGBTQ identity.

Particularly, gender-sexuality alliances or gay-straight alliances (GSAs), school-based support clubs for adolescents from various sexuality, gender, and racial identities, have been researched extensively and have consistently reported greater wellbeing for LGBTQ adolescents compared to adolescents in schools without GSAs (Poteat et al., 2018). However, LGBTQ-POC adolescents reported feeling out of place and feeling uncomfortable in GSAs as LGBTQ-POC adolescents were bashed and silenced by LGBTQ White adolescents due to the lack of racial-ethnic inclusivity (e.g., lack of culturally inclusive LGBTQ role models, exclusive and negative reactions toward ethnic foods during GSA gatherings; Endo, 2021). Therefore, as GSAs have been shown to function as both an advocacy and support network to connect LGBTQ adolescents and address inequalities in schools (Poteat et al., 2018), school staff who act as advisors for GSAs can be equipped to not only support their LGBTQ students but also support and take into consideration the needs of LGBTQ-POC students.

A promising model in educational practice that be further the inclusivity and reflexivity of GSAs for LGBTQ-POC students is the relational health model. The relational health model frames the importance of authenticity and relatability in the environment. Authenticity, in this sense, is defined as relationships that foster a sense of freedom to be genuine in one’s identity and expression (Gamarel et al., 2014). As LGBTQ-POC students feel uncomfortable and are unable to be authentic and develop their sense of self, GSAs can incorporate the relational health model by creating a space for mutual engagement. A space for mutual engagement, characterized by mutual involvement, commitment, and sensitivity to each other in the social space can be set up through a co-creation of social contracts (i.e. rules). Everyone will be required to follow the social contracts to create a respectful space appreciative of the diversity within the LGBTQ student population. However, Poteat and Scheer (2016) found that school advisors were unable to both address issues of sexuality and gender identity and race and ethnicity. They reported feeling the lack of training to address multiple forms of oppression though they understand the importance of GSAs to support and guide all LGBT adolescents in discussion and actions that span beyond LGBT issues and include intersectional issues of race-ethnicity and race-based discrimination (Poteat and Scheer, 2016).

The analysis points to the need for developmental researchers to account for intersectionality during identity development and how Erikson’s stages of psychosocial development may not account for *emerging adults*’ struggles in intersectional identity exploration and consolidation. Additionally, there is a need for stakeholders in schools and researchers in the field of LGBTQ studies, education, and developmental psychology, to expand the research and practice of supporting LGBTQ adolescents in schools to include voices of LGBTQ-POC adolescents and how intersectionality can complicate adolescents’ acceptance of their identity within safe school spaces and entering the expectations and pre-conceived notions that they will face as they develop into LGBTQ-POC emerging adults in the larger LGBTQ community.

## Data Availability Statement

The raw data supporting the conclusions of this article will be made available by the authors, without undue reservation, to any qualified researcher.

## Ethics Statement

Ethical review and approval was not required for the study on human participants in accordance with the local legislation and institutional requirements. The patients/participants provided their written informed consent to participate in this study.

## Author Contributions

The author confirms being the sole contributor of this work and has approved it for publication.

## Conflict of Interest

The author declares that the research was conducted in the absence of any commercial or financial relationships that could be construed as a potential conflict of interest.
